# The Great Desideratum

**Published:** 1896-10

**Authors:** E. W. Berridge


### THE GREAT DESIDERATUM.

We need a complete Repertory of Characteristics. Many years ago Dr. E. J. Lee commenced one, and the chapters published—Mind, Head, and Vertigo—have never been surpassed. It is said that Dr. Lee's health prevents him from completing his work. If so, will he not hand the MSS. over to some other physicians for immediate publication? The glory will still be his, and his alone. But if this cannot be done, let other homœopaths take up the work, and produce a new Repertory arranged on the same plan. Whenever a second edition of the Mind and Head chapters is called for, I can supply a goodly number of additions.

E. W. Berridge, M. D.

